# Interprofessional education-relevant accreditation standards in Canada: a comparative document analysis

**DOI:** 10.1186/s12960-021-00611-1

**Published:** 2021-05-13

**Authors:** Mohammad Azzam, Anton Puvirajah, Marie-Andrée Girard, Ruby E. Grymonpre

**Affiliations:** 1 Curriculum Studies and Studies in Applied Linguistics, Faculty of Education, Western University, London, ON Canada; 2 Anesthesiology and Pain Medicine Department, Faculty of Medicine, University of Montreal, Montreal, QC Canada; 3 Health Hub: Politics, Organizations and Law, Montreal, QC Canada; 4 Faculty of Law, University of Montreal, Montreal, QC Canada; 5 College of Pharmacy, Rady Faculty of Health Sciences, University of Manitoba, Winnipeg, MB Canada

**Keywords:** Interprofessional education, Interprofessional collaborative practice, Health professions accreditation, Document analysis

## Abstract

**Background:**

Increasing evidence suggests that sustainable delivery of interprofessional education (IPE) has the potential to lead to interprofessional collaborative practice (IPCP), which in turn has the potential to lead to enhanced healthcare systems and improved patient-centered care health outcomes. To enhance IPE in Canada, the Accreditation of Interprofessional Health Education (AIPHE) project initiated collaborative efforts among accrediting organizations of six health professions to embed IPE language into their respective accreditation standards. To further understand the impact of the AIPHE project, this study evaluated the accountability of the IPE language currently embedded in Canadian health professions’ accreditation standards documents and examined whether such language spanned the five accreditation standards domains identified in the AIPHE project.

**Methods:**

We conducted a comparative content analysis to identify and examine IPE language within the “accountable” statements in the current accreditation standards for 11 Canadian health professions that met our eligibility criteria.

**Results and discussion:**

A total of 77 IPE-relevant accountable statements were identified across 13 accreditation standards documents for the 11 health professions. The chiropractic, pharmacy, and physiotherapy documents represented nearly 50% (38/77) of all accountable statements. The accountable statements for pharmacy, dentistry, dietetics, and nursing (registered) spanned across three-to-four accreditation standards domains. The remaining nine professions’ statements referred mostly to “Students” and “Educational program.” Furthermore, the majority of accreditation standards documents failed to provide a definition of IPE, and those that did, were inconsistent across health professions.

**Conclusions:**

It was encouraging to see frequent reference to IPE within the accreditation standards of the health professions involved in this study. The qualitative findings, however, suggest that the emphasis of these accountable statements is mainly on the students and educational program, potentially compromising the sustainability and development, implementation, and evaluation of this frequently misunderstood pedagogical approach. The findings and exemplary IPE-relevant accountable statements identified in this paper should be of interest to all relevant stakeholders including those countries, where IPE accreditation is still emerging, as a means to accelerate and strengthen achieving desired educational and health outcomes.

**Supplementary Information:**

The online version contains supplementary material available at 10.1186/s12960-021-00611-1.

## Background

Increasing evidence suggests that interprofessional collaborative practice (IPCP) ultimately leads to enhanced recruitment and retention of healthcare professionals, improved patient-centered care health outcomes, and reduced healthcare costs, thereby resulting in improved efficiency and quality of healthcare services [1–4]. The Centre for the Advancement of Interprofessional Education (CAIPE) defines interprofessional education (IPE) as “occasions when members or students of two or more professions learn with, from and about each other to improve collaboration and the quality of care and services” ([5], p. 1). It is postulated that successful and sustainable delivery (development, implementation, and evaluation) of interprofessional education (IPE) has the potential to lead to meaningful IPCP involving collaboration-ready health professional^1^ graduates [7–9]. As such, the World Health Organization (WHO, [10–15]), among others, has been promoting IPE as an important and innovative pedagogical approach to ultimately improving healthcare services and outcomes by addressing the global workforce crisis [4].

**Fig. 1:**
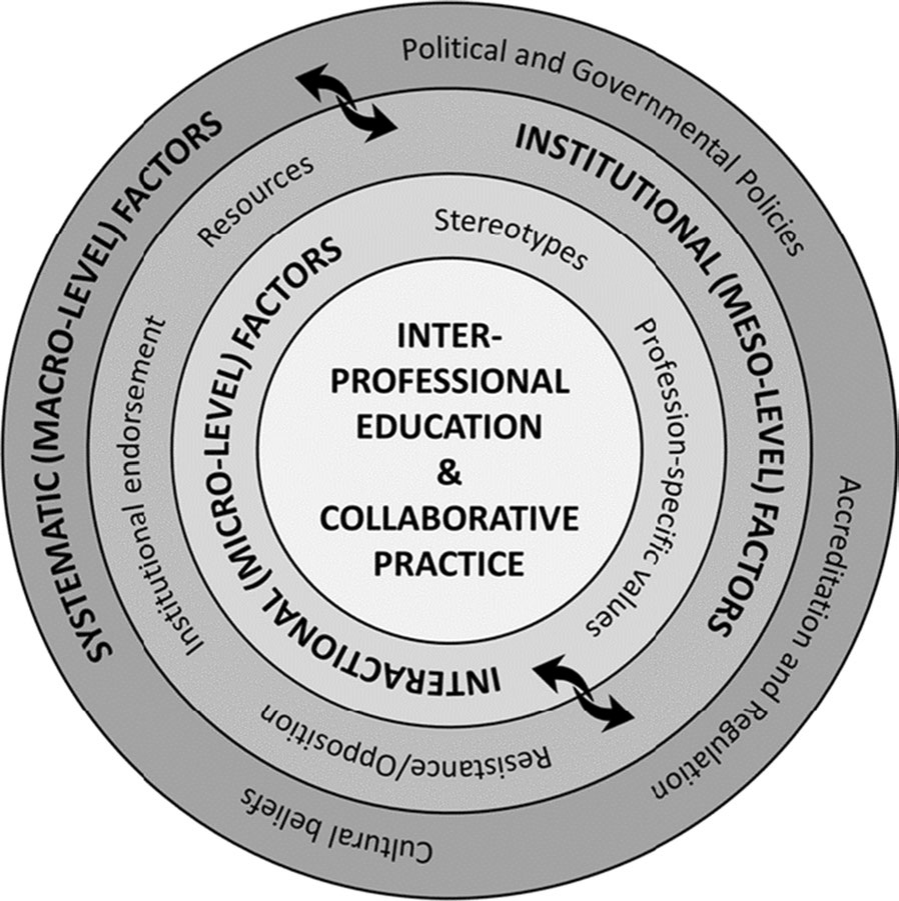
Visual representation of the factors that influence delivery of interprofessional education and collaborative practice. Adapted from the D’Amour framework [16]

The sustainable delivery of both IPE and IPCP, however, is complex, requiring interactions and influences among various micro-, meso-, and macro-level factors (Fig. 1) within and between both academic and healthcare systems [16]. Simultaneous, purposeful and deliberate consideration of these factors is important for sustainable delivery of IPE and IPCP and involves the adoption of common IPE language across health professions; the use of theoretical underpinnings to guide the delivery of IPE; scholarship that informs decision-making and continuous quality improvement of IPE; and the adoption of a collaborative approach to incorporating IPE language in the accreditation and regulation^2^ of the health professions [17].

The presence of IPE language in the accreditation mechanisms (macro-level) can only be significant if it ensures academic programs are accountable and responsive to sustainable delivery of IPE [19, 20]. Furthermore, the WHO’s National Health Workforce Accounts (NHWA) system, specifically Indicator 3-06 [13], is a significant global driver of IPE accreditation. Through enabling IPCP, IPE accreditation is a small, albeit significant, step towards achieving several education and health-related Sustainable Development Goals [21] aimed at ensuring inclusive learning opportunities and promoting well-being for all. IPCP also addresses two of the WHO’s triple billion targets: “1 billion more people better protected from health emergencies” and “1 billion more people enjoying better health and well-being” ([14], p. 1). Despite these efforts and initiatives to influence IPE accreditation and, in turn, the sustainable delivery of IPE and IPCP, integration of IPE in the accreditation mechanisms of the health professions remains fragmented globally [22]—even in countries, where IPE accreditation is relatively sophisticated (e.g., Australia, Canada, the United States).

Within the Canadian context, analyses of the accreditation standards documents for six Canadian health professions revealed that, in 2005, only pharmacy explicitly addressed IPE in their accreditation standards [23, 24]. At the time, Curran et al. [23, 24] concluded that accreditation mechanisms in Canada neither promoted nor fostered IPE. A similar comparative analysis of ten health professions in the United States found that, with the exception of pharmacy, there was an inadequate emphasis on IPE culture as well as an overall lack of collaborative efforts by accrediting organizations in the United States to adopt common IPE language [25]. Similarly, a recent review in Australia demonstrated inconsistencies and inadequacies in standards that held academic programs accountable to providing evidence of IPE [26]. The findings from these studies [23–26] raise concerns that even when IPE language exists within the accreditation standards, not holding academic programs accountable to those standards may lead to minimal sustainable delivery of IPE and ultimately lead to graduating a health workforce that is inadequately prepared for IPCP.

A significant turning point for Canada in terms of IPCP was the Interprofessional Education for Collaborative Patient-centered Practice (IECPCP) initiative (2004–2011) funded ($21 million) by the Government of Canada. A portion of these funds were used to support the establishment of the Canadian Interprofessional Health Collaborative (CIHC), which among its many deliverables included the now globally recognized *National Interprofessional Competency Framework* [27]. This framework, which aligned with the WHO’s *Framework for Action* [11], identified six interprofessional competency domains: (1) Interprofessional communication; (2) Patient-centred care; (3) Role clarification; (4) Team functioning; (5) Interprofessional conflict resolution; and (6) Collaborative leadership, to inform the interprofessional knowledge, skills, and dispositions that are required by all health professionals to work collaboratively. The IECPCP initiative further funded the two-phase Accreditation of Interprofessional Health Education (AIPHE) project (2007–2011) [28, 29], making Canada the first country in the world to use a collaborative approach to developing and embedding IPE language spanning the five accreditation standards domains [29] into the accreditation standards of six health professions: medicine, nursing, occupational therapy, pharmacy, physiotherapy, and social work (Table 1).

**Table 1:** Accreditation standards domains identified in the AIPHE project [29]

Domain	Description
Organizational commitment	Organizational commitment refers to that administrative structures and processes, preferably at the level of the Vice President’s Office and/or deanship, must foster the development, implementation, and evaluation of interprofessional education
Faculty	Faculty members must be supported, encouraged, and prepared to facilitate the development, implementation, and evaluation of interprofessional education
Students	Students must understand the significance of interprofessional education and demonstrate proficiency in interprofessional competencies
Educational program	Educational programs within and across faculties must share a common understanding of IPE and facilitate the development, implementation, and evaluation of interprofessional education throughout the learning continuum for all students
Resources	The human, material, and financial resources that enable the development, implementation, and evaluation of interprofessional education must be supplied

*AIPHE* Accreditation of Interprofessional Health Education

A more recent case study [30] of the Canadian accreditation standards documents for the same six health professions involved in the AIPHE project [28, 29] found IPE language within the documents for all six professions. This case study, however, neither systematically evaluated the “accountability” of such IPE language nor examined the extent to which such language addressed the accreditation standards domains identified in the AIPHE project [29]. Building on this case study, the intention of the present study was to understand the quality and accountability of IPE-relevant accreditation standards across the health professions. It is expected that this research would be a significant step towards understanding the impacts of IPE on IPCP and ultimately patient health outcomes.

## Methods

The present study used comparative content analysis [31] to evaluate the accountability of the IPE language currently embedded in 11 Canadian health professions’ accreditation standards documents and examine whether such language spanned the five accreditation standards domains identified in the AIPHE project [29].

### Identification of health professions

Initially, one author (MAG) researched for existing federal regulatory organizations in addition to laws that regulate professions in each provincial (local) jurisdiction. In so doing, we identified 42 health professions that are regulated in at least one province in Canada (Table 2). Note that we excluded the three Canadian territories from this study as their regulations are linked to other jurisdictions.

**Table 2:** Regulated health professions in Canada (*N* = 42)

1. Acupuncture	22. Nursing (Practitioner)
2. Audiology	23. Nursing (Psychiatric Practitioner)
3. Auxiliary nursing care	24. Nutrition
4. Cardiology technique	25. Occupational therapy
5. Chinese traditional medicine	26. Optical/optician
6. Chiropractic	27. Optometry
7. Counseling therapy	28. Orthotics/prosthetics
8. Dental assistance	29. Paramedicine
9. Dental hygiene	30. Pharmacy
10. Dental technique	31. Pharmacy technique
11. Dental therapy	32. Physiotherapy
12. Dentistry	33. Podiatric Surgery
13. Dietetics	34. Podiatry
14. Homeopathy	35. Psychology
15. Laboratory technique	36. Radiology technique
16. Massage therapy	37. Respiratory therapist
17. Medicine	38. Sexology
18. Midwifery	39. Social Work
19. Multiple techniques	40. Specialists in audiological prostheses
20. Naturopathy	41. Speech Therapy
21. Nursing (registered)	42. Technique in radiation oncology

Next, we focused our query on regulated health professions for feasibility of undertaking this study and consistency with existing practices and research literature on IPE and IPCP. In Canada, the regulation of the health professions is overseen and legally mandated by provincial governments; as some regulated health professions are regulated in all ten provinces, the academic programs of these regulated health professions undergo accreditation by federal organizations. This arrangement allows accreditation to be fully operationalized and applied consistently nationwide. To ensure consistency in selecting the health professions, we used the following three eligibility criteria: (1) the health profession must have at least one federal accrediting organization; (2) the health profession must be regulated across all provinces; and (3) accreditation must be mandatory for entry-to-practice or program recognition by all the provincial regulatory bodies.

Of the 42 health professions in Canada, only 11 met our eligibility criteria: chiropractic, dentistry, dietetics, medicine, nursing (registered), occupational therapy, optometry, pharmacy, physiotherapy, psychology, and social work (Table 3). In addition, medicine itself can be further subdivided into undergraduate medicine and postgraduate specialty medicine, which includes family medicine and all other medical specialties. We address family medicine and other specialty medicine separately in our presentation here. Hereafter, we use the profession name as opposed to the name of the profession’s accrediting organization for simplicity purposes.

**Table 3:** Eligible health professions (*N* = 11) and accrediting organizations

Profession	Accrediting Organization
Chiropractic	Canadian Federation of Chiropractic Regulatory and Education Accrediting Boards (CFCREAB)
Dentistry	Commission on Dental Accreditation of Canada (CDAC)
Dietetics	Partnership for Dietetic Education and Practice (PDEP)
Medicine	
Family medicine	College of Family Physicians of Canada (CFPC)
Specialty medicine	Canadian Residency Accreditation Consortium (CanRAC)
Undergraduate medicine	Committee on Accreditation of Canadian Medical Schools (CACMS)
Nursing (registered)	Canadian Association of Schools of Nursing (CASN)
Occupational therapy	Canadian Association of Occupational Therapists (CAOT)
Optometry	Accreditation Council on Optometric Education (ACOE)
Pharmacy	Canadian Council for Accreditation of Pharmacy Programs (CCAPP)
Physiotherapy	Physiotherapy Education Accreditation Canada (PEAC)
Psychology	Canadian Psychological Association (CPA)
Social work	Canadian Association for Social Work Education (CASWE)

### Locating the accreditation standards documents

Except for dentistry, we located and retrieved the current accreditation standards documents in October 2020 through an online search on their respective organizations’ official websites. Dentistry’s current accreditation standards document was obtained by directly contacting the Commission on Dental Accreditation of Canada (CDAC).

We identified 13 accreditation standards documents [32–44] (Table 4) for the 11 health professions included in this study. Three of these documents [39, 40, 42] were relevant to medicine. We also noted that the Canadian Psychological Association (CPA) independently discusses its two clinical designation streams (Clinical, Counselling, and School Psychology [CCSP] and Clinical Neuropsychology [CNP]) in the same document [38], each involving separate didactic and internship components.

**Table 4:** Current accreditation standards documents (*N* = 13) for eligible health professions (*N* = 11)

Chiropractic
Canadian Federation of Chiropractic Regulatory and Education Accrediting Boards. Standards for accreditation of Doctor of Chiropractic programmes. Canadian Federation of Chiropractic Regulatory and Education Accrediting Boards; 2011. Available from: http://www.chirofed.ca/english/pdf/Standards-for-Accreditation-of-Doctor-of-Chiropractic-Programmes.pdf. Accessed 18 Oct 2020.
Dentistry
Commission on Dental Accreditation of Canada. Accreditation requirements for Doctor of Dental Surgery (DDS) or Doctor of Dental Medicine (DMD) programs. Commission on Dental Accreditation of Canada; 2013.
Dietetics
Partnership for Dietetic Education and Practice. Accreditation standards for dietetic education programs in Canada. Partnership for Dietetic Education and Practice; 2014. Available from: https://www.pdep.ca/library/Accreditation-Policies-and-Standards/PDEP-Accreditation-Standards-for-Dietetic-Educatio.aspx. Accessed 17 Oct 2020.
Family medicine
College of Family Physicians of Canada. Standards of accreditation for residency programs in family medicine. College of Family Physicians of Canada; 2018. Available from: https://portal.cfpc.ca/ResourcesDocs/uploadedFiles/_Shared_Elements/Documents/20180701_RB_V1.2_ENG.pdf. Accessed 14 Oct 2020.
Nursing (registered)
Canadian Association of Schools of Nursing. CASN accreditation programs standards. Canadian Association of Schools of Nursing; 2014. Available from: https://www.casn.ca/wp-content/uploads/2014/12/2014-FINAL-EN-Accred-standards-March-311.pdf. Accessed 14 Oct 2020.
Occupational Therapy
Canadian Association of Occupational Therapists. Academic accreditation standards and self-study guide. Canadian Association of Occupational Therapists; 2019. Available from: https://caot.in1touch.org/uploaded/web/Accreditation/CAOT%20Accreditation%20Self%20Study%20Guide%202017%20English%20rv%202019.pdf. Accessed 17 Oct 2020.
Optometry
Accreditation Council on Optometric Education. Accreditation manual: Professional optometric degree programs. Accreditation Council on Optometric Education; 2019. Available from: https://www.aoa.org/AOA/Documents/Education/ACOE/OD_Manual_%2008_2019_PDF.pdf. Accessed 18 Oct 2020.
Pharmacy
Canadian Council for Accreditation of Pharmacy Programs. Accreditation standards for Canadian first professional degree in pharmacy programs. Canadian Council for Accreditation of Pharmacy Programs; 2017. Available from: http://ccapp-accredit.ca/wp-content/uploads/2016/01/Accreditation-Standards-for-Canadian-First-Professional-Degree-in-Pharmacy-Programs.pdf. Accessed 17 Oct 2020.
Physiotherapy
Physiotherapy Education Accreditation Canada. PEAC accreditation standards: 2012 including essential concepts. Physiotherapy Education Accreditation Canada; 2012. Available from: https://peac-aepc.ca/pdfs/Accreditation/Accreditation%20Standards/PEAC%20Standards%202012%20with%20essential%20concepts%20FINAL.pdf. Accessed 17 Oct 2020.
Psychology
Canadian Psychological Association. Accreditation standards and procedures for doctoral programmes and internships in professional psychology. Canadian Psychological Association; 2011. Available from: https://cpa.ca/docs/File/Accreditation/Accreditation_2011.pdf. Accessed 18 Oct 2020.
Social Work
Canadian Association for Social Work Education. Standards for accreditation. Canadian Association for Social Work Education; 2014. Available from: https://caswe-acfts.ca/wp-content/uploads/2013/03/CASWE-ACFTS.Standards-11-2014-1.pdf. Accessed 18 Oct 2020.
Specialty medicine
Canadian Residency Accreditation Consortium. General standards of accreditation for residency programs. Canadian Residency Accreditation Consortium; 2020. Available from: http://www.canrac.ca/canrac/canrac/documents/general-standards-accreditation-for-residency-programs-e.pdf. Accessed 14 Oct 2020.
Undergraduate medicine
Committee on Accreditation of Canadian Medical Schools. CACMS standards and elements: Standards for accreditation of medical education programs leading to the M.D. degree. Committee on Accreditation of Canadian Medical Schools; 2019. Available from: https://www.cacmscafmc.ca/sites/default/files/documents/CACMS_Standards_and_Elements_AY_2020-2021.pdf. Accessed 14 Oct 2020.

In addition, if the accreditation standards documents referenced and required compliance and adherence by health professions academic programs to separate, supporting documents [45–47] (Table 5), we analyzed those documents as well. One statement, for example, stated, “The curriculum is student/intern centred and based on achieving the ‘Integrated Competencies for Dietetic Education and Practice (ICDEP)’” ([43], p. 9). If the ICDEP document [46] discussed such “integrated competencies” in terms of IPE, we deemed the statement to be relevant.

**Table 5:** Supporting documents (*N* = 3) for current accreditation standards documents (*N* = 13)

Dietetics
Partnership for Dietetic Education and Practice. The Integrated Competencies for Dietetic Education and Practice (ICDEP). Partnership for Dietetic Education and Practice; 2013. Available from: https://www.pdep.ca/library/Accreditation-Policies-and-Standards/PDEP-ICDEP-2013-.aspx. Accessed 28 Oct 2020.
Family medicine
Shaw E, Oandasan I, Fowler N. CanMEDS Family Medicine 2017: A competency framework for family physicians across the continuum. College of Family Physicians of Canada; 2017. Available from: https://portal.cfpc.ca/resourcesdocs/uploadedFiles/Resources/Resource_Items/Health_Professionals/CanMEDS-Family-Medicine-2017-ENG.pdf. Accessed 28 Oct 2020.
Specialty medicine
Frank JR, Snell L, Sherbino J. CanMEDS 2015 Physician Competency Framework. Royal College of Physicians and Surgeons of Canada; 2015. Available from: http://canmeds.royalcollege.ca/uploads/en/framework/CanMEDS%202015%20Framework_EN_Reduced.pdf. Accessed 28 Oct 2020.

### Categorization of eligible statements

**Fig. 2:**
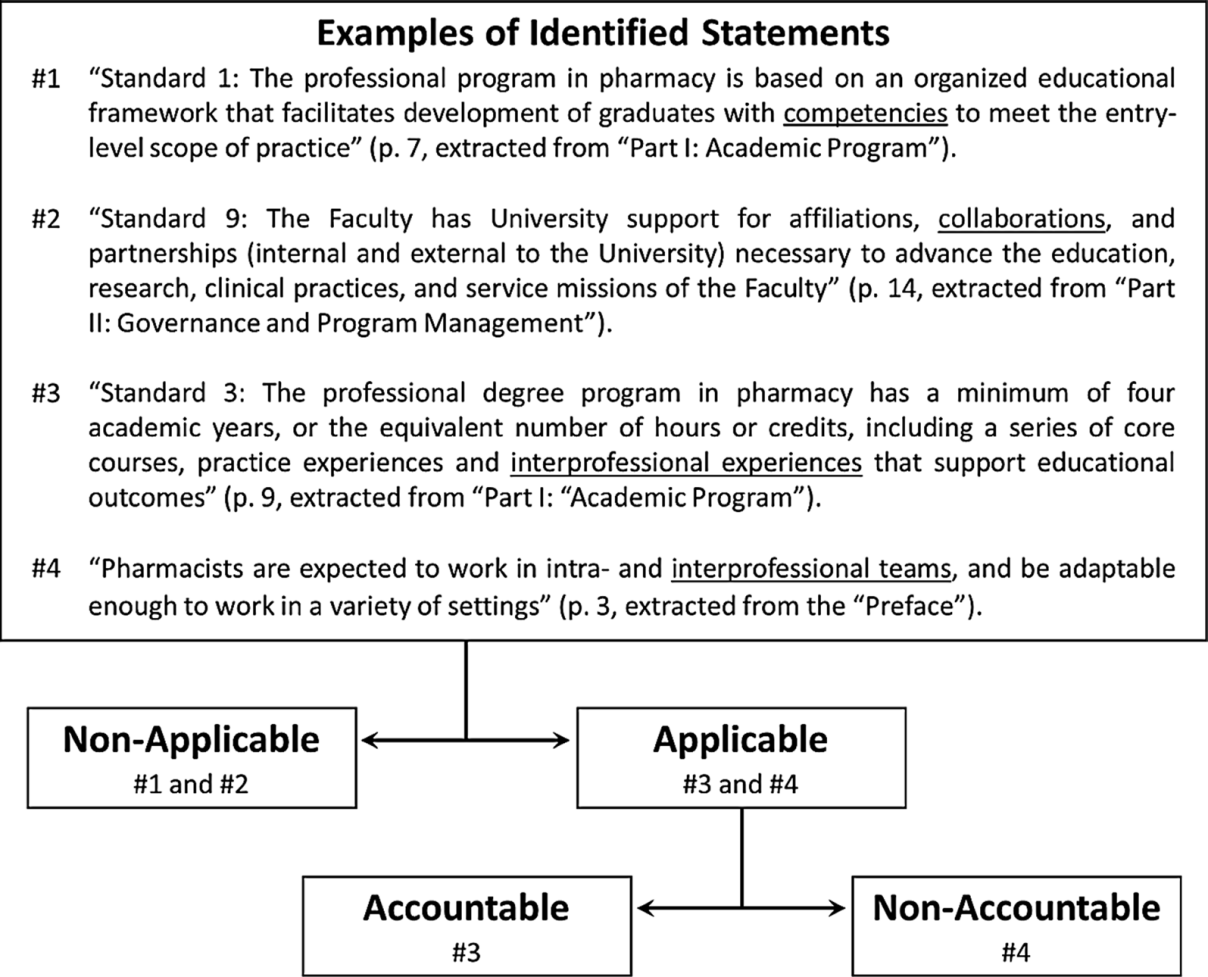
Examples of the categorization of eligible statements from the pharmacy accreditation standards document [36]. A “non-applicable” statement refers to a statement that was identified as potentially being relevant to IPE but upon analysis was found to be not relevant. For instance, we cannot unquestionably determine that statement #1 referred to interprofessional competencies. Similarly, statement #2 generally described collaborative intra-institutional and inter-institutional endeavors, with no specific reference to IPE. An “applicable” statement encompassed an explicit IPE expression. An “accountable” statement was one to which the accrediting organizations held their respective academic programs accountable. Statement #3 expressed this definition clearly. A “non-accountable” statement was one to which the accrediting organizations could not hold their respective academic programs accountable, such as statement #4, which was located in the preface of the document

The unit of analysis in this study was comprised of all potential IPE-relevant statements in the 13 accreditation standards documents [32–44]. Employing a categorization scheme used in previous studies [25, 26], these statements were categorized into one of three categories: (1) non-applicable; (2) applicable, but non-accountable; and (3) applicable and accountable (Fig. 2). A “non-applicable” statement refers to a statement that we identified as potentially being relevant to IPE but upon analysis was found to be not relevant, whereas an “applicable” statement encompasses an explicit IPE-relevant expression. Applicable statements were then further categorized as either non-accountable or accountable. An “accountable” statement was one to which the accrediting organizations held their respective programs accountable. Accountable statements were typically located within IPE-relevant accreditation standards and criteria statements. Furthermore, a “non-accountable” statement was one to which the accrediting organizations could not hold their respective programs accountable. Non-accountable statements were typically located in titles and section headings, introductory or summative sections, flowcharts, footnotes, glossaries, and appendices. Finally, applicable statements that either made generic reference to “examples of evidence” or were noted to be “exemplary” or “desirable” were categorized as non-accountable as such statements were not mandatory and to which accrediting organizations cannot hold their respective programs accountable.

### Analysis procedures

Initially, two authors (MA and AP) undertook a 75 min “training session,” where they independently read and hand searched the pharmacy accreditation standards document [36] and highlighted all statements containing potential IPE-relevant language. Next, they independently coded each identified statement according to the described categorization scheme. Furthermore, using the keywords “collaboration,” “interprofessional,” and “interprofessional education,” the second author (REG) and her research assistant (TP) both conducted an electronic search of the same document to confirm that no eligible statements were overlooked. Once comfortable with the coding process and the categorization scheme, we repeated this procedure for the remaining documents.

We ran Fleiss’ κ to determine the inter-rater reliability of the categorization between MA and AP using IBM SPSS Statistics for Windows, Version 27 [48]. We reported mean estimations along with 95% confidence intervals (CI). Interpretation of inter-rater reliability was as follows: *x* ≤ 0.20, poor; 0.20 < *x* ≤ 0.40, fair, 0.40 < *x* ≤ 0.60 moderate; 0.60 < *x* ≤ 0.80, good; 0.80 < *x* ≤ 1.00, very good [49]. Afterwards, MA and AP compared and discussed the results of their coding process and negotiated and resolved any discrepancies that arose.

Furthermore, MA and REG independently and deductively coded the accountable statements against the five accreditation standards domains identified in the AIPHE project [29] and negotiated and resolved any discrepancies that arose. Finally, we determined whether a definition of “interprofessional education” was specified, or at a minimum was acknowledged, in the accreditation standards documents.

## Results

For the 11 health professions included in this study, 13 accreditation standards documents [32–44] and three supporting documents [45–47] were retrieved. A total of 208 statements within these documents were deemed potentially IPE-relevant. Inter-rater reliability was substantial; concordance between the two raters was 90.38% (188/208). Fleiss’ κ illustrated that there was very good agreement between the two raters, κ = 0.854 (95% CI, 0.757–0.951), *p* < 0.005. Discrepancies in the remaining 20 statements were resolved through discussion until consensus was reached.

Final categorization of the 208 statements resulted in 77 (37.02%) non-applicable statements, 54 (25.96%) non-accountable statements, and 77 (37.02%) accountable statements. The non-accountable statements were found in the Introduction/Preface (*n* = 6), Table of Contents (*n* = 7), headings/subheadings (*n* = 1), within non-mandatory (e.g., examples, exemplary, and desirable) standards (*n* = 32), glossaries (*n* = 4), and appendices (*n* = 3). A perfunctory examination of the 13 accreditation standards documents revealed that chiropractic, pharmacy, and physiotherapy documents represented nearly 50% (38/77) of all accountable statements, whereas the optometry document contained no accountable statements. Table 6 illustrates the number of categorized statements for each health profession.

**Table 6:** Categorization of statements potentially relevant to interprofessional education

Profession	Non-applicable	Non-accountable	Accountable
Chiropractic	0	1	11
Dentistry	3	1	6
Dietetics	2	0	9
Family medicine	14	3	4
Nursing (registered)	13	4	7
Occupational therapy	2	3	3
Optometry	0	0	0
Pharmacy	10	29	13
Physiotherapy	9	6	14
Psychology (CCSP program)	1	1	0
Psychology (CCSP internship)	4	2	0
Psychology (CNP program)	1	0	1
Psychology (CNP internship)	3	2	0
Social work	2	0	1
Specialty medicine	12	1	7
Undergraduate medicine	1	1	1
Total of 208 (%)	77 (37.02%)	54 (25.96%)	77 (37.02%)

*CCSP* Clinical, Counselling, and School Psychology, *CNP* Clinical Neuropsychology. A “non-applicable” statement refers to a statement that was identified as potentially relevant to IPE but indeed was not. A “non-accountable” statement did not require action on part of the health professions academic programs. An “accountable” statement required action on part of the health professions academic programs and to which the accrediting organizations held their respective programs accountable

**Fig. 3:**
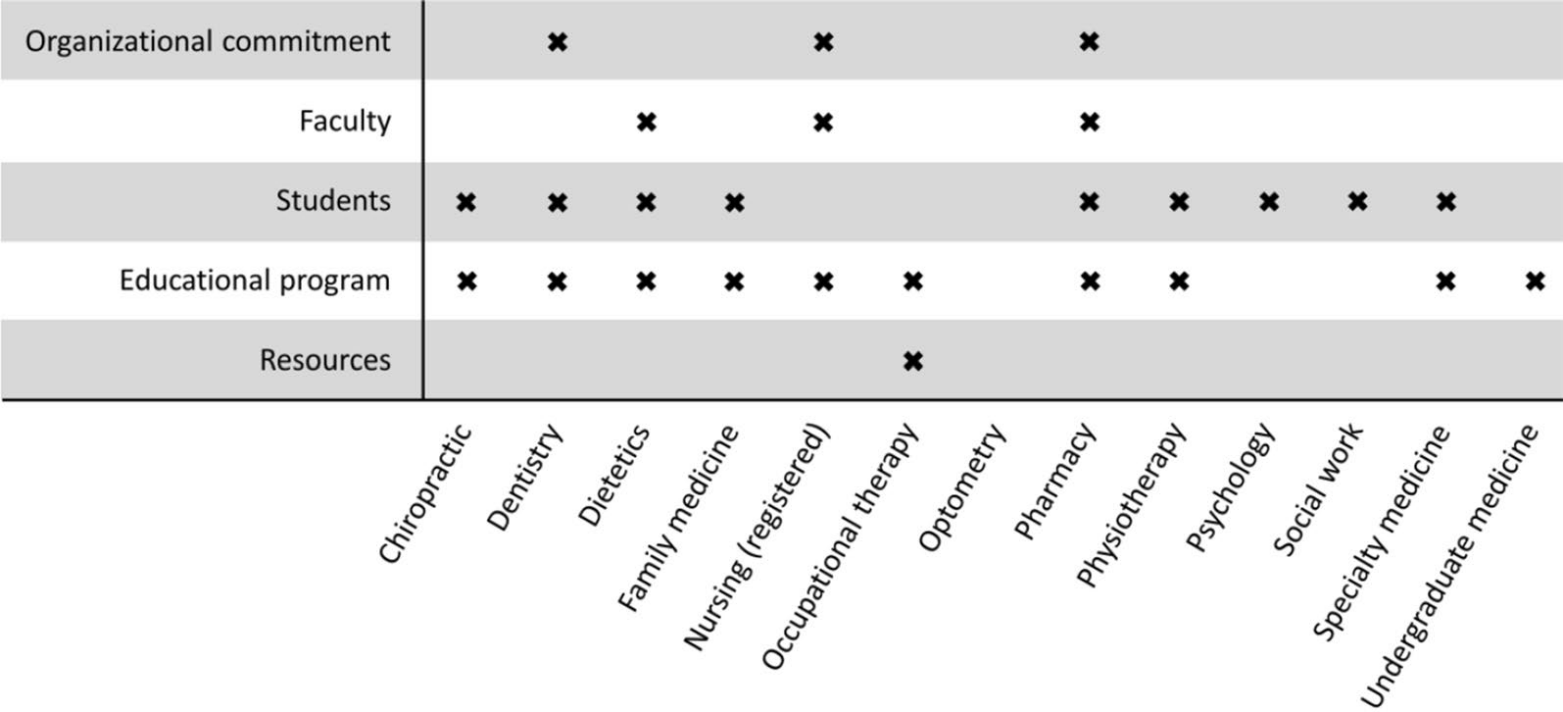
Coding of accountable statements across the five accreditation standards domains [29]. For psychology, only the CCSP program incorporated IPE language in their only accountable statement

Figure 3 illustrates which of the five accreditation standards domains identified in the AIPHE project [29] were addressed in the accountable statements for each health profession. The most common domains across health professions (*n* = 13) were “Educational program” (*n* = 10; 76.92%) and “Students” (*n* = 9; 69.23%). The accountable statements for pharmacy alone spanned four of the five accreditation standards domains, whereas the accountable statements for dentistry, dietetics, and nursing (registered) spanned three of these domains. The domains covered by the other health professions spanned from zero to two domains. Furthermore, it was noted that for eight of the health professions (dentistry, dietetics, medicine (family and specialty subdivisions), nursing [registered], occupational therapy, pharmacy, physiotherapy, and psychology [CNP program]), the accountable statements that addressed “Educational program” made reference to practice-based learning.

Table 7 illustrates a sampling of exemplary accountable statements extracted from the accreditation standards documents spanning the five accreditation standards domains identified in the AIPHE project [29]. See Additional file 1 for all 77 accountable statements.

**Table 7:** Exemplary accountable statements across accreditation standards domains [29]

Domain	Exemplary accountable statement
Organizational commitment	“The University has integrated and endorsed the concept of interprofessional education and collaboration in practice” ([36], p. 16)
Faculty	“Preceptors are academically and experientially qualified for their role in assisting interns to achieve the ICDEP” ([43], p. 13)
Students	“Students should be exposed to the principles of interprofessional collaboration for the provision of patient care” ([41], p. 30)
Educational program	“The program provides opportunities for learners to develop knowledge, skills, and attitudes in using relevant information, communication technology, critical thinking, and clinical reasoning, in the delivery of collaborative client-centered care” ([35], p. 25)
Resources	“A report that documents the IPE activities and experiences integrated in the occupational therapy program. The report should describe the program offerings, and include considerations of space, human and learning resources required to deliver IPE ([34], p. 19)

*ICDEP* Integrated Competencies for Dietetic Education and Practice, *IPE* interprofessional education

Finally, only four of the 13 accreditation standards documents (for family medicine, occupational therapy, physiotherapy, and specialty medicine) included a definition of IPE. Family and specialty medicine cited their definitions from the CanMEDS framework [45, 47], which in turn cited the Royal College of Physicians and Surgeons of Canada (RCPSC) [50]. Furthermore, the documents for both occupational therapy and physiotherapy cited adapted versions of the globally accepted definition from CAIPE [5]. Of significance, the definitions from family and specialty medicine excluded a key component for IPE—learning “with, from and about” each other.

## Discussion

In this study, we undertook an assessment of IPE language embedded within the 13 current accreditation standards documents for 11 regulated health professions in Canada. Our analysis revealed that the number of accountable IPE-relevant accreditation standards contained in these documents ranged from zero (for optometry, psychology [CCSP program; CCSP internship; and CNP internship]) to over ten (for chiropractic, pharmacy, and physiotherapy). Nonetheless, we caution against judging the quality of IPE-relevant accreditation standards strictly by the number of accountable statements in each document. The AIPHE project [28, 29] was careful to not mandate a particular structure or content to the standards. Rather, guiding principles involved the adoption of a common lexicon, focusing on the common mandate of IPE as opposed to professional differences, and allowing for flexibility in how each accrediting organization developed their standards and collected their evidence.

That being said, the presence of IPE language contained within “accountable” statements in accreditation standards documents is critical as it provides the accrediting organizations the authority to look for evidence of IPE in the academic programs they accredit and hold those programs accountable and responsive to meeting these standards. The absence of IPE language in the accreditation standards documents for optometry and psychology (CCSP program; CCSP internship; CNP internship) implies a lack of incentive for these programs to deliver sustainable, evidence-based IPE.

Of greater significance than the absolute number of accountable statements, however, was our finding that the accountable standards for most professions spanned only two (“Students” and “Educational program”) of the five accreditation standards domains identified in the AIPHE project [29]. Only the accountable statements for pharmacy spanned four domains (“Organizational commitment,” “Faculty,” “Students,” and “Educational program”). It was reassuring, however, to see that in addressing the “Educational program” domain, eight of the 13 accreditation standards documents reviewed in this study referenced practice-based IPE in one or more accountable statements. To date, there has been limited global emphasis on advancing practice-based interprofessional learning compared to didactic learning. The hypothesis is that practice-based accreditation standards would incentivize innovative practice-based IPE [30].

While emphasis on “Educational program” and “Students” is of obvious importance, a lack of emphasis on the other three domains potentially hinders sustainable delivery of IPE within academic institutions [17]. The D’Amour framework [16] illustrated the micro-, meso-, and macro-level factors that must be addressed when delivering IPE within the educational system. In addition to emphasis on educational programs and student learning, the framework identifies “organizational commitment” and the allocation of adequate “resources” as essential meso-level factors that influence the sustainable delivery of IPE. Furthermore, the framework emphasizes the key roles of “faculty” and faculty development as critical to offering theoretically grounded and evidence-based IPE. At the micro-level, faculty are essential to fostering a culture that enables positive interprofessional learning interactions as opposed to a “hidden curriculum” that fuels stereotypes, miscommunication, and mutual distrust among students of different health professions [51, 52], which, in turn, generates healthcare professionals who are unable to effectively collaborate within an interprofessional team. Hence, incorporating IPE language across the five pillars of accreditation standards domains is imperative. That being said, data supporting the impact of accreditation on the quality of educational programs and further the impact on graduates’ performance in clinical environments, and ultimately on the quality of patient care is complex and our current understanding is limited [53]. Blouin [53] has recently proposed a framework of outcome markers to guide future research in this emerging area.

Furthermore, it was noteworthy that inductive analyses of the accountable statements within ten health professions’ accreditation standards documents in the United States [25] found that the IPE language also spanned five major domains (“IPE inclusion in mission and goals,” “IPE responsibilities of the Dean,” “allocation of budgetary and fiscal resources to IPE,” “IPE inclusion in curricular programs and defined learning outcomes,” and “student competencies”). These categories generally paralleled four of the five accreditation standards domains identified in the AIPHE project [29], with only “Faculty” not addressed in the United States’ analysis. Hence, it seems that the scope of IPE-relevant standards determined by both Canadian and the United States’ collaborative efforts were closely aligned, in essence serving as a validation of the domain categories, and further underscoring the recommendation that implementation of sustainable, evidence-based IPE innovations requires accreditation standards that span all five domains.

It was also noteworthy that in this study, with the exception of family medicine, occupational therapy, physiotherapy, and specialty medicine, most accreditation standards documents failed to provide definitions for IPE—a finding similar to Bogossian and Craven’s findings in Australian accreditation standards documents [26]. The globally accepted definition of IPE is that from CAIPE [5]. To ensure delivery of evidence-based and theoretically informed IPE, it is imperative to have a common understanding across all relevant stakeholders of what IPE is and what it is not [28]. For example, an interprofessional panel presentation to a group of students from various health professions, is not IPE, as this activity comprises one-way exchange of knowledge with no interaction among learners from different professions and, therefore, no opportunities for them to learn “with, from and about” each other. Therefore, the lack of a definition of IPE in most accreditation standards documents and the missing text “with, from and about” from the definitions from family and specialty medicine are concerning and need to be addressed.

The greatest limitation to this study was exclusion of the 31 health professions that did not meet our eligibility criteria. Our findings are limited to the 11 health professions reviewed and can neither be representative nor generalizable to other professions without further investigation. Furthermore, most accreditation standards documents failed to provide definitions for IPE; thus, we could only assume that when they referenced IPE language, they were all referring to IPE as defined by CAIPE [5]. Finally, this study examined the accreditation standards documents for IPE-relevant statements to which accrediting organizations can hold their respective academic programs accountable, but did not examine types of evidence provided by the academic programs in meeting these standards across all five accreditation standards domains [29]. A team of CIHC researchers is currently conducting a national survey to address this research objective.

## Conclusions

This research provides an informative update on the incorporation of IPE into the accreditation standards of a large number of health professions in Canada. Though evidence of the real-world impact of such standards is scant, and measurement and attribution of team performance is quite challenging, this study can be seen as providing early evidence of the relative value of IPE as judged by national leadership in a large number of health professions.

The AIPHE project’s [28, 29] approach that aimed to embed IPE language into the accreditation standards for six Canadian health professions academic programs appears to have directly or indirectly influenced several other Canadian health professions not involved in AIPHE. The standards for chiropractic and psychology are cited as having a publication year of 2011―the same year as the AIPHE guidelines, thereby suggesting that such professions may not have had the opportunity to review their guidelines and incorporate adequate IPE language before they last published an update to their standards. More interestingly is that the professions (e.g., optometry and undergraduate medicine) with the least thematic coverage are among those that have more recently updated their standards.

Furthermore, this study found that IPE language in the accountable statements within a majority of health professions’ accreditation standards documents were mostly relevant to “Students” and “Educational program.” The emphasis within the “Educational program” on practice-based IPE was especially noteworthy. The authors suggest these standards could be even more comprehensive and explicit. The lack of emphasis on “Resources” and “Organizational commitment” raises concerns regarding sustainable delivery of IPE within any given institution. Furthermore, the lack of emphasis on “Faculty” raises concerns about the quality of IPE being offered. To enable evidence-based IPE and IPCP, it is our recommendation that all relevant stakeholders including accrediting organizations and educational and healthcare systems in Canada and elsewhere, adopt a common definition of IPE—the most widely accepted definition provided by CAIPE [5]. We assert that the adoption of the findings and exemplary IPE-relevant accountable statements highlighted in this paper will be of global relevance, especially for those countries where accreditation and more specifically, accreditation of IPE, are still emerging.

## Supplementary Information

**Additional file 1**. All accountable statements, spanning the five accreditation standards domains identified in the AIPHE project [29].

## Data Availability

The data sets supporting the conclusions of this article are available from the corresponding author upon reasonable request.

## Abbreviations

ACOE: Accreditation Council on Optometric Education
AIPHE: Accreditation of Interprofessional Health Education
CACMS: Committee on Accreditation of Canadian Medical Schools
CAIPE: Centre for the Advancement of Interprofessional Education
CanRAC: Canadian Residency Accreditation Consortium
CAOT: Canadian Association of Occupational Therapists
CASN: Canadian Association of Schools of Nursing
CASWE: Canadian Association for Social Work Education
CCAPP: Canadian Council for Accreditation of Pharmacy Programs
CCSP: Clinical, Counselling, and School Psychology
CDAC: Commission on Dental Accreditation of Canada
CFCREAB: Canadian Federation of Chiropractic Regulatory and Education Accrediting Boards
CFPC: College of Family Physicians of Canada
CIHC: Canadian Interprofessional Health Collaborative
CNP: Clinical Neuropsychology
CPA: Canadian Psychological Association
ICDEP: Integrated Competencies for Dietetic Education and Practice
IECPCP: Interprofessional Education for Collaborative Patient-centered Practice
IPCP: Interprofessional collaborative practice
IPE: Interprofessional education
NHWA: National Health Workforce Accounts
PDEP: Partnership for Dietetic Education and Practice
PEAC: Physiotherapy Education Accreditation Canada
RCPSC: Royal College of Physicians and Surgeons of Canada
WHO: World Health Organization

## Footnotes

1 According to Statistics Canada, “health professionals” are those who “are primarily concerned with diagnosing and treating health problems in humans and animals and with providing related services” ([6], p. 15). Hence, any profession that specializes in physical health, mental health/social services, dental health, or ocular health can be classified as a health profession.

2 “Accreditation” and “regulation” within the context of education are not the same. The WHO defines “accreditation” as the “formal process by which a recognized body … assesses and recognizes that a health care organization meets applicable pre-determined and published standards. Accreditation standards are usually regarded as optimal and achievable and are designed to encourage continuous improvement efforts within accredited organizations” ([12], p. 1). Whereas the WHO defines “regulation” as the “imposition of external constraints upon the behaviour of an individual or an organization to force a change from preferred or spontaneous behaviour” ([12], p. 14). In other words, the regulation of a health profession typically involves regulatory mandates issued by a regulatory authority (oftentimes, a professional body or government entity) to maintain high standards of practice for the profession. Non-compliance of a regulatory mandate may lead to administrative or penal consequences, as the objective of regulation is the control of unwanted behaviours, contrary to accreditation, which is the promotion of best practices [18].
